# The case for transitioning to living systematic reviews in rapidly evolving clinical fields

**DOI:** 10.1097/MS9.0000000000005645

**Published:** 2026-09-08

**Authors:** Zohaib Hassan, Ayesha Tariq, Sabeen Tahir, Rejina Chhetri, Janu Chhetri, Marina Kc

**Affiliations:** a Punjab Medical College, Faisalabad, Punjab, Pakistan; b Federal Medical and Dental College, Islamabad, Pakistan; c Nepalgunj Medical College, Kohalpur, Banke, Nepal; d Department of Medicine, Parbat Provincial Hospital, Kushma, Nepal

*Dear Editor*,

Traditional systematic reviews have long played a major role in the clinical literature and the development of clinical guidelines^[^1^]^. However, as medicine advances and new data continue to yield divergent findings, there is an urgent need for living systematic reviews, in which new evidence is iteratively added and updated. Such an approach facilitates immediate guideline updates following the publication of new results. This would enhance clinical practice and ensure the population receives treatment based on the most recent evidence, fulfilling the primary objective of research: improved patient care^[^2^]^. Furthermore, the current latency between the initial search and publication of a systematic review often exceeds a year, delaying the incorporation of evidence into standard guidelines.

There is an imperative to practice evidence-based medicine rather than the “legacy medicine” that currently predominates. Static systematic reviews – and the clinical guidelines derived from them – often become outdated within 12–18 months of publication^[^3^]^. This is frequently due to large-scale trials reporting significant new findings, some demonstrating drastic changes in the efficacy or safety profiles of a drug or procedure. Consequently, physicians continue to prescribe based on obsolete guidelines, potentially endangering patient safety^[^4^]^. While maintaining a living systematic review requires greater effort, this investment is justified to prevent the “cost of inaction” and ensure healthcare remains aligned with the most recent evidence.

To bridge this gap, medical journals need to change. We urge editors to encourage this shift through specific pathways for “living” content and peer-review processes that support iterative updates. While the current system is built for static papers, we must prioritize more responsive models of evidence to ensure the evidence we synthesize keeps pace with what is happening at the bedside.

Despite their significant clinical advantages, living systematic reviews pose hurdles. They require continuous surveillance, funding, and institutional support. Methodological concerns include rerunning meta-analyses after adding new trials, which may increase the risk of false positives due to repeated significance testing. Peer reviewers are already overburdened, making continuous incremental updates difficult to manage. The PRISMA reporting guidelines for living systematic reviews also remain a major hurdle.

## Data Availability

No datasets were generated or analyzed during the preparation of this Letter to the Editor.

## Research registration unique identifying number (UIN)

Not applicable for Letter to the Editor as per journal.
